# Distinct endotypes of steroid-resistant asthma characterized by IL-17A^high^ and IFN-γ^high^ immunophenotypes: Potential benefits of calcitriol

**DOI:** 10.1016/j.jaci.2015.01.026

**Published:** 2015-09

**Authors:** Emma S. Chambers, Alexandra M. Nanzer, Paul E. Pfeffer, David F. Richards, Peter M. Timms, Adrian R. Martineau, Christopher J. Griffiths, Christopher J. Corrigan, Catherine M. Hawrylowicz

**Affiliations:** a MRC and Asthma UK Centre for Allergic Mechanisms in Asthma, King's College London, London, United Kingdom; b Asthma UK Centre for Applied Research, Centre for Primary Care and Public Health, Blizard Institute, Queen Mary, University of London, London, United Kingdom; c Homerton University NHS Foundation Trust, London, United Kingdom

**Keywords:** Asthma, steroid resistant, steroid sensitive, glucocorticoids, IL-17A, vitamin D, BAL, Bronchoalveolar lavage, GR-β, Glucocorticoid receptor β, 25(OH)D, 25-Hydroxyvitamin D, 1,25(OH)_2_D3, 1,α25-Dihydroxyvitamin D, ROC, Receiver operating characteristic, SR, Steroid resistant, SS, Steroid sensitive

## Abstract

**Background:**

A small population of patients with severe asthma does not respond to glucocorticoids (steroid resistant [SR]). They have high morbidity, highlighting an urgent need for strategies to enhance glucocorticoid responsiveness.

**Objective:**

We investigated the immunologic differences between steroid-sensitive (SS) and SR asthmatic patients and the effect on immunophenotype of oral calcitriol treatment because it has been previously shown to beneficially modulate the clinical response to glucocorticoids in patients with SR asthma.

**Methods:**

CD8-depleted PBMCs were isolated from 12 patients with SS and 23 patients with SR asthma and cultured for 7 days with anti-CD3 and IL-2 with or without dexamethasone. Cytokine production was assessed in supernatants by using the Cytometric Bead Array. Patients with SR asthma were subsequently randomized to oral calcitriol or placebo therapy, and identical studies were repeated.

**Results:**

Patients with SR asthma produced significantly increased IL-17A and IFN-γ levels compared with those in patients with SS asthma, although it was evident that cells from individual patients might overproduce one or the other of these cytokines. Production of IL-17A was inversely and production of IL-13 was positively associated with the clinical response to prednisolone. Oral calcitriol, compared with placebo, therapy of the patients with SR asthma significantly improved dexamethasone-induced IL-10 production *in vitro* while suppressing dexamethasone-induced IL-17A production. This effect mirrored the previously demonstrated improvement in clinical response to oral glucocorticoids in calcitriol-treated patients with SR asthma.

**Conclusions:**

IL-17A^high^ and IFN-γ^high^ immunophenotypes exist in patients with SR asthma. These data identify immunologic pathways that likely underpin the beneficial clinical effects of calcitriol in patients with SR asthma by directing the SR cytokine profile toward a more SS immune phenotype, suggesting strategies for identifying vitamin D responder immunophenotypes.

Asthma is an inflammatory lung disease characterized by airways hyperresponsiveness and remodeling and is one of the most common long-term medical conditions. The majority of asthmatic patients achieve control of their disease with β_2_-adrenergic agonists and glucocorticoids (steroids) and therefore have steroid-sensitive (SS) asthma.^1^ However, there is a proportion of patients who do not show improvement in symptoms and lung function after compliant treatment with inhaled steroids, oral steroids, or both; these patients with steroid-resistant (SR) asthma experience considerable ill health and impart a considerable financial burden on the health care system.

Asthma is a heterogeneous disease, and phenotype-specific therapies have been suggested to enhance the likelihood of therapeutic success in patients relatively refractory to conventional therapy.^2^ Recently, significant effort has been directed at defining severe asthma and, in particular, its endotypes or phenotypes.^3^ A number of newer biological agents are currently being tested in clinical trials, and although therapeutic benefit has been observed with some of them, they appear to target small subsets of patients.^4-9^ Furthermore, they are expensive to manufacture and administer. On the other hand, steroid-sparing or enhancing strategies for refractory patients remain relatively unexplored.

Asthma historically was characterized as being a CD4^+^ T_H_2-mediated disease with increased production of IL-4, IL-13, and IL-5. More recently, these studies have been refined to identify clinical and immunologic subtypes (phenotypes and endotypes, including immunophenotypes). For example, there is established evidence of an IL-17A^high^ phenotype in patients with severe asthma. Subgroups of patients with severe asthma have been demonstrated to have increased concentrations of the proinflammatory cytokine IL-17A in sputum and bronchoalveolar lavage (BAL) fluid.^10-13^ Additionally, IL-17A production has been correlated with airways hyperresponsiveness.^14^ Although T_H_17 cells have been shown to be important for defense against fungal and bacterial lung infections, a critical balance between this and an association with various immune pathologies, including autoimmune disease, appears to exist. Studies in mice suggest an association between steroid resistance and T_H_17-mediated disease: adoptive transfer of T_H_17 cells resulted in increased concentrations of chemokines and granulocyte colony-stimulating factor in the BAL fluid of mice with severe combined immunodeficiency, and treatment with dexamethasone resulted in increased neutrophil infiltration but no improvement in airways hyperresponsiveness.^15^ More recently, we showed that blood T-cell IL-17A expression was increased 7-fold in patients with SR asthma compared with that seen in patients with SS asthma.^16^ Additionally, we found that although the synthetic glucocorticoid dexamethasone did not inhibit IL-17A production, calcitriol (1,α25-dihydroxyvitamin D [1,25(OH)_2_D3]), the active form of vitamin D, significantly inhibited IL-17A production in a glucocorticoid-independent manner.^16^ Additionally SR asthma has been associated with increased expression of the transcriptionally inactive glucocorticoid receptor β (GR-β),^17^ whereas increased GR-β expression has been reported in response to exogenous IL-17A and IL-23 *in vitro*, an effect that was more prominent in asthmatic patients than in healthy control subjects.^18,19^ Together, these studies constitute a firm platform for implicating IL-17A in the pathogenesis of SR asthma.

There is a large body of epidemiologic data suggesting that vitamin D insufficiency, as defined by the serum 25-hydroxyvitamin D (25[OH]D) concentration, is strongly associated with impaired respiratory health,^20^ risk of asthma,^21,22^ asthma severity, and refractoriness to current therapy.^23-27^ We have previously shown that patients with SR asthma have a defective T-cell IL-10 response to dexamethasone *in vitro*^28^ and that this could be restored by 1,25(OH)_2_D3.^29^ These data, along with the epidemiologic evidence, prompted us to design a proof-of-concept, placebo-controlled clinical trial assessing whether treatment with calcitriol could restore clinical responsiveness to a standard 2-week trial of oral prednisolone in patients with SR asthma. Indeed, in this trial we showed that treatment with calcitriol resulted in significant improvement in steroid responsiveness in these patients.^30^

The aim of the present study was to use PBMC samples from the patients in this trial to further assess the immunophenotypes of SS and SR asthma at baseline and after calcitriol therapy in the patients with SR asthma to identify potential immunophenotypes of steroid refractoriness and also the likely effect of treatment with calcitriol.

## Methods

### Subjects

Asthmatic patients receiving step 3 or 4 therapy according to the British Thoracic Society guidelines and receiving optimized care by tertiary care respiratory physicians were recruited and provided written informed consent (ethics 08/H0804/84). All patients had a prebronchodilator FEV_1_ of less than 80% of predicted value and documented airway variability of greater than 12% after 400 μg of short-acting bronchodilator and had undergone detailed assessment to exclude a diagnosis other than asthma and comorbidities affecting asthma control. Patients had not received oral glucocorticoids (steroids) for at least 4 weeks before the study. Patients receiving immunotherapy, smokers, or patients with a respiratory tract infection or asthma exacerbation during the 4 weeks before enrollment were excluded.

Steroid resistance was defined as less than 10% improvement in baseline prebronchodilator FEV_1_ after a 14-day course of oral prednisolone (40 mg/1.73 m^2^/d; Wockhardt UK, Wrexham, United Kingdom) in eligible patients. Routine spirometry was measured before and after the course of prednisolone by using a PC-based spirometer and software (WinspiroPRO Medical International Research, Rome, Italy).

**Fig E1:**
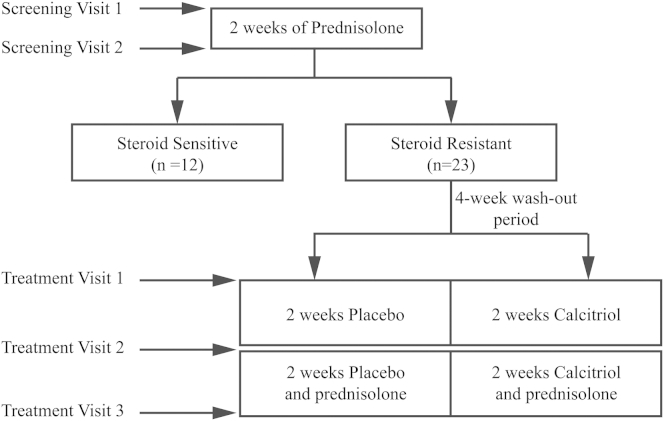
Clinical trial schematic. Patients with severe asthma were recruited and given prednisolone at screening visit 1 and returned 2 weeks later at screening visit 2 when patients with SS asthma were excluded from the trial and patients with SR asthma were retained. After a 4-week washout period, the patients with SR asthma were randomly assigned to receive calcitriol or placebo for 1 month (treatment visit 1). After 2 weeks (treatment visit 2), the patients were started on additional oral prednisolone and then returned 2 weeks later for the final visit (treatment visit 3). Lung function was assessed at all clinical trial visits except treatment visit 1. CD8-depleted PBMCs were assessed from peripheral blood collected at screening visit 1 and treatment visit 3.

Participants who were identified as having SR asthma were randomized to receive either 0.25-μg calcitriol soft capsules (Rocaltrol; Roche, Mannheim, Germany) or organoleptically identical lactose placebo generated in house (Pharmacy Production Unit, St Thomas' Hospital NHS Trust, London, United Kingdom) twice daily for 4 weeks after a 4-week washout period (full details of the clinical trial study outline and patient details can be found in the article by Nanzer et al^30^; see Fig E1 in this article's Online Repository at www.jacionline.org for a study schematic). After 2 weeks of calcitriol or placebo treatment, patients were given a second course of oral prednisolone identical to the first while calcitriol or placebo was continued. Spirometry was performed at the beginning and end of this second course of oral prednisolone, as before.

### Flow cytometry

The following antibodies were used for *ex vivo* phenotyping of peripheral blood obtained from asthmatic donors: CD3, CD4, CD8, and CD19 (SK7, RPA-T4, RPA-T8, and HIB19, respectively; BD Biosciences, Oxford, United Kingdom). Red blood cells were lysed after staining with BD FACS lysing solution; the samples were subsequently assessed on a FACSCalibur (BD Biosciences). Absolute and differential blood leukocyte counts were performed routinely with an LH750 hematology analyzer (Beckman Coulter, Brea, Calif) and analyzed in conjunction with flow cytometric data to calculate cell numbers.

### Cell isolation and culture

Human PBMCs were isolated, as previously described.^29^ Briefly, CD8-depleted PBMCs were obtained by means of negative selection with CD8^+^ Dynabeads (Invitrogen, Paisley, United Kingdom). Cells (1 × 10^6^ cells/mL) were cultured in RPMI (containing 10% FCS, 2 mmol/L l-glutamine and 50 μg/mL gentamicin) and stimulated with plate-bound anti-CD3 (1 μg/mL, OKT-3) plus 50 U/mL recombinant hIL-2 (Eurocetus, Harefield, United Kingdom) in the presence or absence of dexamethasone at indicated concentrations (Sigma-Aldrich, Gillingham, United Kingdom) and/or 10 ng/mL hIL-4 in a 24-well plate for 7 days. There was no significant difference in cellular viability under all culture conditions (data not shown). Where indicated, after this initial 7-day period, cells were harvested and readjusted to the same density of 1 × 10^6^/mL viable cells and then cultured for a further 48 hours with plate-bound anti-CD3 and IL-2 alone in 48-well plates, after which supernatants were harvested for cytokine analysis.

### Cytokine analysis

IL-17A, IL-10, IFN-γ, and IL-13 concentrations in culture supernatants were measured by using the Cytometric Bead Array (BD Biosciences), according to the manufacturer's protocol. The lower limit of detection for each analyte was 1.5 pg/mL.

### Statistics

Data were assessed for equivalence to a Gaussian distribution and equality of variance, after which the appropriate parametric or nonparametric test was performed (see individual figure legends) with GraphPad Prism 6 software (GraphPad Software, La Jolla, Calif). Differences were considered significant at the 95% confidence level. Data are presented as means, with error bars representing 95% CIs.

## Results

**Fig E2:**
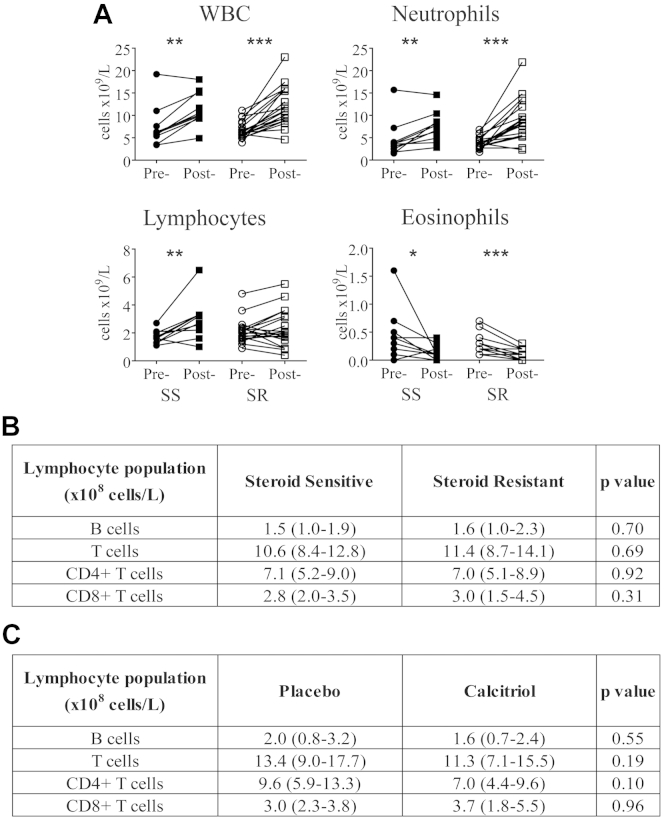
Peripheral leukocyte counts. **A,** Peripheral blood total and differential leukocyte counts in the patients with SS asthma *(solid symbols)* and patients with SR asthma *(open symbols)* before and after 2 weeks of prednisolone. **B** and **C,** Total B cells (CD19^+^), T cells (CD3^+^), and CD4^+^ and CD8^+^ T cells were assessed by using *ex vivo* flow cytometric analysis of peripheral blood from screening visit 1 of both patients with SS and those with SR asthma (Fig E2, *B*), as well as in the same patients with SR asthma separated based on their assignment to calcitriol or placebo treatment (Fig E2, *C*). Data were assessed by using the Wilcoxon matched-pairs statistical test (Fig E2, *A*) or the Mann-Whitney *U* statistical test (Fig E2, *B* and *C*). *P ≤ .05, **P < .01, and ***P < .001.

To investigate the immunologic phenotypes of SS and SR asthma, we recruited patients with moderate-to-severe asthma who were defined as having either SS or SR asthma based on their changes in lung function after 2 weeks of therapy with oral prednisolone at pharmacodynamically uniform dosages (for a clinical trial schematic, see Fig E1).^30^ The patients with SS and SR asthma were similar in terms of demographics, mean body mass index, mean equivalent inhaled glucocorticoid dosages, and mean FEV_1_ at baseline. The only clinical difference between patients in the 2 groups was their changes in lung function after oral prednisolone (mean ΔFEV_1_ percent predicted); the patients with SS asthma showed a significant improvement (from 56.0% [95% CI, 47.4% to 64.6%] to 70.8% [95% CI, 62.6% to 79.0%], *P* < .0001), whereas the patients with SR asthma did not (from 61.3% [95% CI, 55.3% to 67.3%] to 59.7% [95% CI, 52.8% to 66.5%], *P* = .18; Table I).^30,31^ There was no significant difference in mean peripheral blood eosinophil counts in the patients with SS asthma compared with those in the patients with SR asthma. Oral prednisolone therapy was associated in both groups with a significant increase in mean blood neutrophil counts and a reduction in mean blood eosinophil counts (see Fig E2 in this article's Online Repository at www.jacionline.org).

### Increased expression of IL-17A and IFN-γ in patients with SR asthma compared with patients with SS asthma

Because asthma is believed to be a CD4^+^ T cell–mediated disease, we used CD8-depleted PBMC cultures with T-cell stimulation, as performed in our previous studies,^16,29^ to compare T-cell immunophenotypes in both patients with SS and those with SR asthma. Blood samples for these studies were taken at screening visit 1 (see Fig E1). CD8-depleted PBMCs from both groups were cultured for 7 days with anti-CD3 and IL-2 in the presence or absence of IL-4. After 7 days, cells were washed, recounted, and replated at 1 × 10^6^/mL to allow for any differential changes in the numbers of cells between the groups and stimulated for a further 48 hours. Cytokine protein expression in culture supernatants was then assessed by using the Cytometric Bead Array for IL-17A, a T_H_17 cytokine; IL-13, a T_H_2 cytokine; and IFN-γ, a T_H_1 cytokine, as well as the anti-inflammatory cytokine IL-10. There were no significant differences in *ex vivo* mean numbers of blood T cells (including CD4^+^ and CD8^+^ cells) and B cells in the asthmatic patients classified as having SS or SR asthma (see Fig E2, *B*).

Under these conditions, CD8-depleted PBMCs from the patients with SR asthma produced significantly increased mean concentrations of both IL-17A and IFN-γ compared with the those seen in the patients with SS asthma, although the mean production of IL-10 and IL-13 did not significantly differ. This difference in IFN-γ production between the 2 groups was no longer observed when exogenous IL-4 was included in the culture conditions (Table II). Attempts were made to detect IL-17A in the cultures containing exogenous IL-4, but concentrations were low or undetectable (data not shown). This is in line with earlier studies showing that IL-4 inhibited IL-17A production, whereas anti–IL-4 enhanced IL-17A production from CD4^+^ T cells.^32^ Conversely, IL-4 signaling through the IL-4 receptor is known to enhance IL-10 responses.^33^

### Differential response to the glucocorticoid dexamethasone in patients with SR asthma compared with that in patients with SS asthma

**Fig 1:**
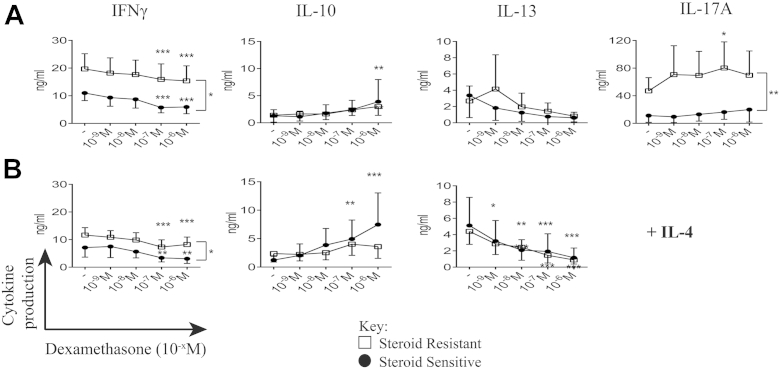
Differential cytokine production by both patients with SS and those with SR asthma in response to dexamethasone *in vitro*. Cytokine production by CD8-depleted PBMCs *(y-axis)* in the presence of dexamethasone (10^−x^ mol/L) *in vitro (x-axis)* stimulated with CD3 plus IL-2 **(A)** and additional IL-4 **(B)** is shown. *Open squares*, Patients with SR asthma; *solid circles*, patients with SS asthma. Data are means and 95% CIs and assessed by using 2-way ANOVA with Dunnett multiple comparisons tests of cytokine production in the presence of different concentrations of dexamethasone compared with no dexamethasone. *P ≤ .05, **P < .01, and ***P < .001.

We next examined whether there were differences in responsiveness to the synthetic steroid dexamethasone *in vitro* between patients with SS asthma and patients with SR asthma that might reflect the differences seen clinically. Cells from the patients with SR asthma compared with those from the patients with SS asthma continued to show increased mean production of IL-17A and IFN-γ across a range of dexamethasone concentrations. Indeed, high concentrations of dexamethasone further significantly increased IL-17A production by cells from the patients with SR asthma, a phenomenon not observed in cells from the patients with SS asthma. Although increased production of IFN-γ by cells from the patients with SR asthma was retained in the presence of dexamethasone, high concentrations of dexamethasone nevertheless inhibited IFN-γ production in both the patients with SS and those with SR asthma. Dexamethasone significantly inhibited IL-13 production by cells cultured in the presence of IL-4 from patients in both groups. Conversely, dexamethasone increased IL-10 production only in cells from the patients with SS asthma and strikingly so in the presence of exogenous IL-4 (*P* = .008), which is in line with our earlier observations (Fig 1, *B*).^28^

### IL-13 and IL-17A production associate positively and negatively with clinical responsiveness to glucocorticoid therapy

**Fig E3:**
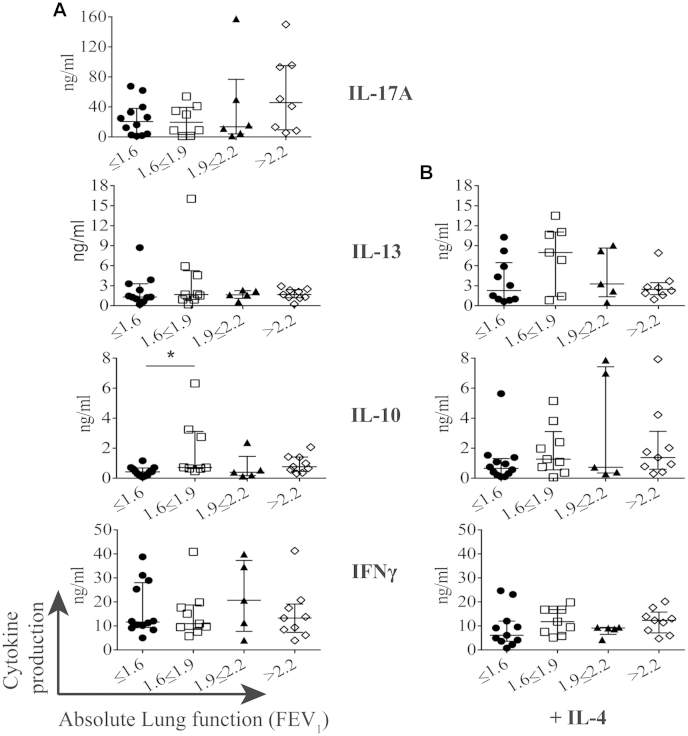
**A** and **B**, Cytokine production does not associate with lung function. Cytokine production from all patients with severe asthma separated according to their absolute lung function at baseline (FEV_1_) is shown. Data were assessed by using Kruskal-Wallis 1-way ANOVA with the Dunn multiple comparison *post hoc* statistical test.

**Fig 2:**
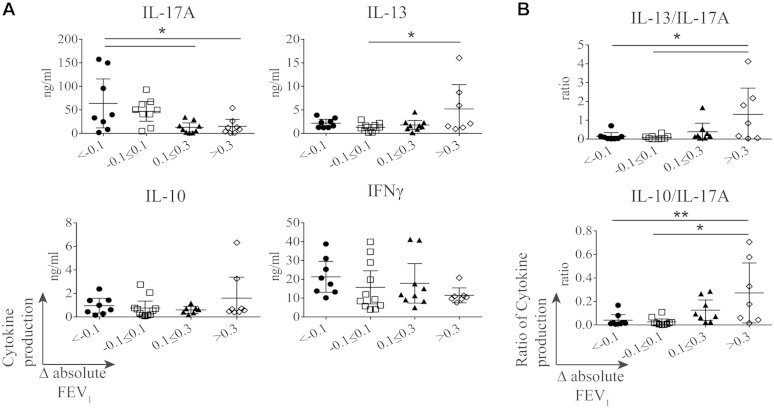
IL-17A is negatively and IL-13 is positively associated with clinical response to 2 weeks of prednisolone. Cytokine production **(A)** and ratio of IL-13/IL-17A and IL-10/IL-17A cytokine production **(B)** separated based on change in absolute lung function in response to 2 weeks of prednisolone (ΔFEV_1_) are shown. Data are means and 95% CIs and assessed by using Kruskal-Wallis 1-way ANOVA with the Dunn multiple comparison *post hoc* statistical test. *P ≤ .05 and **P < .01.

We next investigated whether these observed differences correlated with clinical measurements. Cytokine production was analyzed according to absolute lung function in both the patients with SS and those with SR asthma, and there was no significant association with production of any cytokine (see Fig E3 in this article's Online Repository at www.jacionline.org). However, when the patients with severe asthma were segregated into quartiles according to their change in lung function after 2 weeks of oral prednisolone therapy (ΔFEV_1_ [in liters]), it was apparent that cells from those asthmatic patients in the lowest quartile whose lung function actually decreased with steroid therapy produced a significantly increased mean quantity of IL-17A at baseline compared with those who showed an improvement. There was a similar trend with IFN-γ production, although this was not statistically significant. Conversely, cells from those asthmatic patients who were in the upper quartile of lung function improvement produced a significantly increased mean quantity of IL-13 at baseline. No such association was observed with IL-10 production (Fig 2, *A*), although those patients whose cells produced the highest mean ratios of IL-10/IL-17A, as well as IL-13/IL-17A, were more likely to fall in the upper quartile of the degree in lung function improvement after taking oral prednisolone (Fig 2, *B*).

### Production of IL-17A and IFN-γ predict clinical glucocorticoid responsiveness

**Fig 3:**
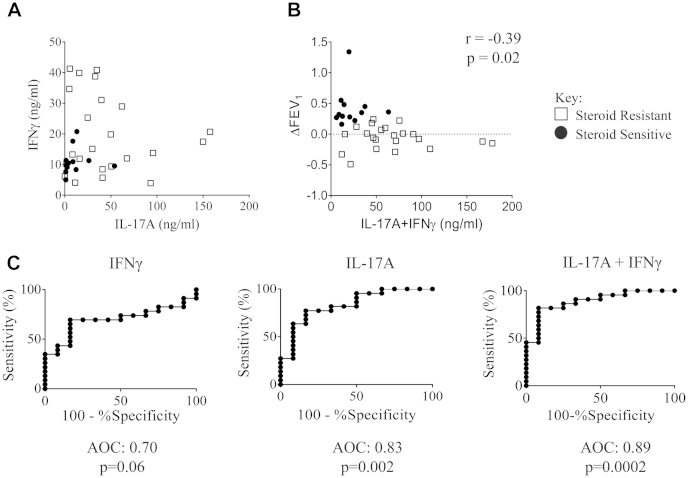
IL-17A and IFN-γ production predict glucocorticoid responsiveness. **A,** Comparison of IL-17A and IFN-γ production in patients with SS asthma *(solid circles)* and patients with SR asthma *(open squares)*. **B,** Combined production of IL-17A and IFN-γ correlated with change in absolute lung function (ΔFEV_1_) in response to 2 weeks of prednisolone, as assessed by using the Spearman rank correlation statistical test. **C,** ROC curves for prediction of steroid resistance based on production of IFN-γ, IL-17A, and their combination.

Because increased production of both IL-17A and IFN-γ by CD8-depleted PBMCs was associated with clinical unresponsiveness to glucocorticoid therapy, we compared production of these cytokines in the entire population of asthmatic patients (both SS and SR asthma). Contrary to our expectations, there was no correlation between overproduction of IL-17A and IFN-γ (Fig 3, *A*), suggesting that both immunophenotypes contribute to corticosteroid responsiveness. Indeed, combined production of both cytokines correlated inversely with change in absolute FEV_1_ after glucocorticoid therapy (Fig 3, *B*). Additionally, we performed analysis of the data in Fig 3, *B*, in the absence of the potential outlier (with ΔFEV_1_ = 1.34), and interestingly, in the absence of this point, the correlation was statistically stronger, with a new *r* value of −0.41 and a *P* value of .018.

Consequently, we analyzed the sensitivity and specificity of IL-17A and IFN-γ production, both separately and in combination, to predict the clinical effect of oral prednisolone therapy in changing FEV_1_. In the case of IL-17A, receiver operating characteristic (ROC) analysis was highly significant (*P* = .002): an IL-17A production threshold of greater than 28.1 ng/mL produced a sensitivity of 63.6% and a specificity of 91.7% for detecting glucocorticoid resistance. In the case of IFN-γ, ROC analysis provided less robust outcomes (*P* = .06): an IFN-γ production threshold of greater than 20.7 ng/mL produced a sensitivity of 34.8% and a specificity of 91.7%. The combination of IL-17A and IFN-γ production proved to be a better test: ROC analysis was highly significant (*P* = .0002), showing that a production threshold of greater than 38.5 ng/mL for the sum of both cytokines predicted clinical glucocorticoid resistance with a sensitivity of 81.8% and specificity of 91.7% (Fig 3, *C*). This degree of sensitivity and specificity is at least comparable with that of noninvasive markers, which have been claimed to predict glucocorticoid-resistant asthma.^34^

### IL-13 production positively correlates with serum 25(OH)D concentrations

**Fig 4:**
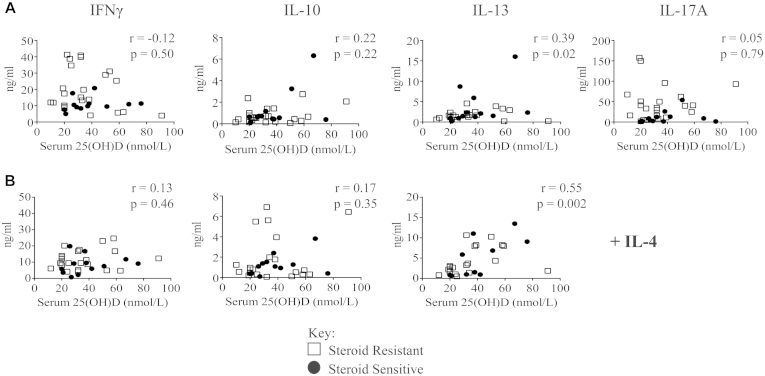
Serum 25(OH)D concentration correlates with IL-13 expression *in vitro*. Cytokine production in cell-culture supernatants was assessed by using the Cytometric Bead Array and correlated with baseline vitamin D status (defined as the serum 25[OH]D concentration). **A,** Cells stimulated with CD3 plus IL-2. **B,** Cells stimulated with addition of IL-4 in culture. *Open circles*, Patients with SR asthma; *solid squares*, patients with SS asthma. Data were assessed by using Spearman rank correlation.

Vitamin D has been reported to inhibit lymphocyte production of IL-17A and IFN-γ *in vitro*^16,29,35^ while enhancing T_H_2 cytokine production.^36^ To examine such possible effects *in vivo*, we next compared cytokine production in all of the study participants with their vitamin D status based on serum 25(OH)D concentrations before calcitriol or placebo therapy. Serum 25(OH)D concentrations did not correlate with production of IL-10, IL-17A, or IFN-γ, but we did observe a significant positive correlation with IL-13 production that was further strengthened in the presence of exogenous IL-4 (Fig 4). After removal of a potential outlier (67 nmol/L; 16 ng/mL), IL-13 still significantly correlated (r = 0.35; *P* = .047) with serum 25(OH)D levels in the cultures without exogenous IL-4.

### Calcitriol therapy of patients with SR asthma reverses induction of IL-17A and augments IL-10 production in response to dexamethasone

**Fig E4:**
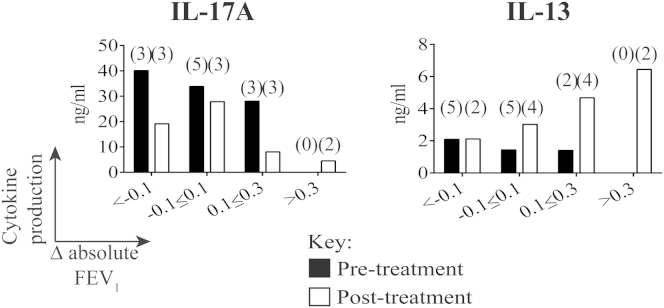
Effects of calcitriol on lung function and IL-13 and IL-17A levels. Cytokine production was separated based on individual changes in absolute lung function (ΔFEV_1_) in response to 2 weeks of prednisolone before (baseline; *black bars*) and after (after 4 weeks of calcitriol and 2 weeks of prednisolone; *white bars*) treatment. Numbers of patients with SR asthma are indicated in parentheses above.

**Fig 5:**
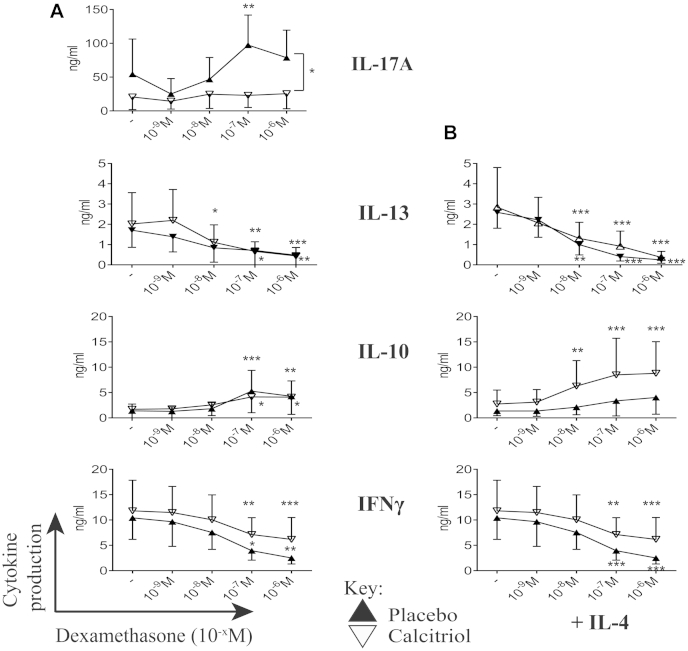
Calcitriol treatment decreases expression of IL-17A in cultures from patients with SR asthma. Cytokine production by CD8-depleted PBMCs from patients with SR asthma in the presence of dexamethasone (10^−x^ mol/L) *in vitro* after 4 weeks of calcitriol or placebo therapy and 2 weeks of oral prednisolone therapy and cells stimulated with CD3 plus IL-2 **(A)** and additional IL-4 **(B)** is shown. *Open triangles*, Calcitriol; *solid triangles*, placebo. Data are means and 95% CIs. Data were assessed by using 2-way ANOVA with Dunnett multiple comparisons tests. *P ≤ .05, **P < .01, and ***P < .001.

In our recent clinical report of the same patients used in the present study, we showed that treatment of our patients with SR asthma with calcitriol compared with placebo improved their clinical responsiveness to glucocorticoid therapy.^30^ We used samples collected from these patients, and notably, no difference was observed between patients with SR asthma subsequently allocated to either placebo or calcitriol treatment at baseline before any treatments with regard to their lymphocyte populations at screening visit 1 (see Fig E2, *C*). We studied these patients to establish the possible effect of oral calcitriol therapy on the immunologic phenotype of peripheral blood cells. We found that after 4 weeks of therapy with calcitriol/placebo and an additional identical 2-week course of oral prednisolone for the final 2 of these 4 weeks (treatment visit 3, see Fig E1), there was significant induction by dexamethasone of IL-17A production in the placebo-treated group, as seen at baseline (screening visit 1). Strikingly, this effect was no longer evident in the calcitriol-treated group (Fig 5, *A*). Furthermore, as predicted from our earlier work,^28,29^ cells from the calcitriol-treated patients with SR asthma now showed a significant increase in IL-10 production in response to dexamethasone, an effect that was most marked in the presence of IL-4 in culture (Fig 5, *B*). Although IL-13 production correlated with serum 25(OH)D concentrations at baseline, as shown above, there was no apparent effect of 4 weeks of calcitriol treatment on production of IL-13 or its inhibition by dexamethasone. Similarly, dexamethasone modestly but significantly inhibited IFN-γ production as before and equivalently in the calcitriol- and placebo-treated patients. Although the numbers are small, further analysis of the calcitriol-treated patients with SR asthma showed that the patients whose improvement in lung function was in the upper quartile in response to the second course of oral prednisolone were those whose cells produced the highest mean quantities of IL-13 and the lowest mean quantities of IL-17A (see Fig E4 in this article's Online Repository at www.jacionline.org).

## Discussion

The data presented in this article demonstrate that there is a significantly higher production of IL-17A and IFN-γ in PBMC cultures from patients with SR asthma compared with those from patients with SS asthma. Notably, these appear to reflect 2 distinct immunophenotypes of SR asthma, an IL-17A^high^ and an IFN-γ^high^ profile, which are largely nonoverlapping. We have previously reported,^30^ and develop here, evidence that glucocorticoids can aggravate the excessive production of proinflammatory T_H_17 by blood lymphocytes in patients with SR asthma, which characterizes a subgroup of these patients. We demonstrate that the levels of IL-17A synthesis in the PBMC cultures negatively and IL-13 synthesis positively correlate with the absolute change in lung function (FEV_1_) after 2 weeks of oral prednisolone treatment in our adult asthma cohort. Our data further support earlier findings that T_H_2^high^ asthma, as characterized by increased IL-13 production, is more likely to be associated with glucocorticoid responsiveness.^37^

The second major finding of this study relates to the capacity of calcitriol to reverse the SR immune phenotype to one more closely aligned to that of patients with SS asthma. Thus the IL-17A^high^ profile is abrogated by calcitriol therapy in a glucocorticoid-independent manner. Conversely, calcitriol therapy also restores the impaired, corticosteroid-induced anti-inflammatory IL-10 response that characterizes patients with SR asthma, extending our earlier findings in this area.^29^ In addition to effects on IL-10 synthesis, calcitriol might reduce the induction of IL-17A through enhancement of a number of regulatory mechanisms, including the inhibitory CD39/adenosine pathway and regulatory T cells.^16,35,38-40^

Several mechanisms have been proposed to contribute to clinical glucocorticoid refractoriness in asthmatic patients at the cellular level, particularly in T cells, including overexpression of proinflammatory transcriptional regulators, such as nuclear factor κB and activator protein 1; increased expression of histone deacetylases, polymorphisms of the *IL10* gene; increased expression of the dominant negative isoform of the GR-β; overexpression of T_H_2 cytokines; and vitamin D insufficiency.^17,24,25,41-44^ More recent evidence also suggests that IL-17A overexpression can be both a marker and a risk factor for severe and SR asthma.^11-14,16^ In this study we confirm the latter association and show an independent association with overexpression of the T_H_1 cytokine IFN-γ. Previous mechanistic studies in autoimmune models of disease have suggested that pathologic IL-17A production is by IL-17A^+^IFN-γ^+^ coexpressing cells,^45-47^ but our data were not consistent with this, rather suggesting separate “immunophenotypes” of SR asthma. It is notable from our study that the IL-17A^high^ phenotype might be enhanced by glucocorticoids in some subjects and that patients with this phenotype were more likely to show deterioration in lung function after oral prednisolone, raising the possibility that oral glucocorticoid therapy is not only unhelpful but also detrimental in some of these patients. Although lymphocyte cultures might not be practical as routine clinical biomarkers, further research might be anticipated to reveal more practical novel biomarkers of IFN-γ^high^ and IL-17A^high^ SR asthma similarly to the development of periostin as a biomarker of T_H_2^high^ asthma.^48^

The mechanisms behind glucocorticoid enhancement of IL-17A production by CD4 cells are not fully understood and represent an area of active research. A recent article described a detailed transcriptional time course during T_H_17 differentiation, suggesting a number of potential candidates for further study.^49^ Previous data from our laboratory suggest that glucocorticoids do not inhibit proliferation of IL-17A–expressing cells; the mechanisms behind these differential effects on proliferation are unknown and currently under investigation. The differences observed in this study are not due to differences in the *ex vivo* baseline populations of the major lymphocyte groups because no differences were observed; however, we cannot rule out there being an enrichment of certain subsets of memory cells. The only difference we have observed previously was in the frequency of FoxP3-positive regulatory T cells,^39^ where we showed that there was a higher frequency of FoxP3-positive T cells in the peripheral blood of patients with SS asthma compared with that seen in patients with SR asthma. However, it seems unlikely that this small population of cells can account for the large differences observed in this study.

It is important to note that a somewhat contradictory scientific literature exists on the capacity of vitamin D to inhibit and/or enhance T_H_2 responses experimentally and in animal models (reviewed by Lange et al^50^). Our data indicate that serum concentrations of 25(OH)D positively correlate with IL-13 synthesis in culture. Importantly, however, administration of oral calcitriol did not lead to enhancement of IL-13 production in culture. Although IL-13 is implicated in asthma pathogenesis, its production is strongly inhibited by glucocorticoids^16,51^; indeed, the T_H_2^high^ asthma phenotype is now widely regarded as a marker of glucocorticoid responsiveness.^37^ Therefore our data support the concept that vitamin D improves responsiveness to glucocorticoids at least partly by altering the immunophenotype away from a glucocorticoid-resistant T_H_17 phenotype toward a more responsive T_H_2 immunophenotype. Thus although calcitriol does not improve lung function in asthma directly, it does improve responsiveness to glucocorticoid therapy.^30^

A limitation to this study was that the data observed were collected from peripheral blood, although we were able to show significant correlations between cytokine production in culture and changes in lung function. Future studies need to be directed at the target organ to gain full insight into changes in cytokine expression in these patients. It would be informative to have studied BAL samples, as well as peripheral blood, providing more information about the tissue of interest. However, bronchoscopy with BAL carries risks and side effects, especially in patients with severe asthma, and therefore was not part of the current study design. One approach we took to counteract this limitation was to perform our peripheral blood cultures in the presence or absence of IL-4 in an attempt to simulate the T_H_2-like environment of the asthmatic bronchial mucosa.^52^ In our previous work we showed that the vitamin D–induced effects on IL-10 were most profound when IL-4 was present in culture.^28,40,53^

Data from several clinical trials investigating the clinical efficacy of vitamin D in asthma are emerging.^30,54-56^ The biggest published trial to date (408 patients randomized) was the VIDA trial. In this placebo-controlled trial patients were supplemented with an initial bolus of 100,000 IU oral vitamin D3 and then subsequently supplemented with 4,000 IU daily. The primary outcome was time to first asthma treatment failure, and no significant improvement in this outcome after vitamin D supplementation was observed. However, the trial also assessed changes in the clinical response to inhaled glucocorticoids, and vitamin D3 supplementation modestly decreased the overall dose of steroid used.^54^ Additionally, a striking reduction in the cumulative number of exacerbations was observed in patients within the treatment arm. Two recent smaller studies showed a more positive role for vitamin D supplementation in asthmatic patients in which vitamin D therapy improved lung function and reduced asthma exacerbations in the treatment arms compared with the placebo arm.^56,57^ Another interesting earlier trial investigated the effect of vitamin D3 supplementation (1,200 IU/d) on seasonal influenza A infection and, despite low numbers, in a secondary outcome measure a significant reduction in the number of asthma attacks in the group that was receiving vitamin D3 was reported.^55^

The studies reported to date have used different forms of vitamin D at different concentrations and dosing regimens over varying periods of time. In several studies many patients were not or were borderline vitamin D deficient, which will affect the efficacy of vitamin D treatment. Clearly, further investigation is required to fully understand the effect of vitamin D treatment on asthma control and prevention. What is unique about our study was the use of the active form of vitamin D, calcitriol, which we have previously shown to improve the SR asthmatic response to 2-week prednisolone therapy. Calcitriol is an attractive short-term adjunct to oral glucocorticoid therapy for severe exacerbations of disease in patients with SR asthma, particularly those already taking maintenance oral glucocorticoids. Although there are concerns about long-term prescription of this form of vitamin D, short-term therapy might be a viable strategy, particularly because expression of Cyp27B1, the catalytic enzyme that converts circulating 25(OH)D into active calcitriol (1,25[OH]_2_D3), is inhibited by glucocorticoids^58^; treatment of patients with SR asthma with oral calcitriol would bypass this effect.

In summary, comparison of immunophenotypes in both patients with SS and those with SR asthma highlights both IL-17A^high^ and IFN-γ^high^ immunophenotypes as independent indicators of glucocorticoid refractoriness and suggests that calcitriol ameliorates the glucocorticoid responsiveness of IL-17A^high^ patients. We believe the data from this study, although from a small cohort, justify further investigation of calcitriol therapy, and comparison with 25(OH)D supplementation in patients with SR asthma identified with biomarkers indicative of nonresponsiveness to glucocorticoids.

**Key messages**

• IL-17A^high^ and IFN-γ^high^ immunophenotypes are indicative of SR asthma.
• Calcitriol potentially improves the clinical response to glucocorticoids through reduction in IL-17A production and enhancement of steroid-induced IL-10.

## Figures and Tables

**Table I:** Patients' characteristics

	Patients with SS asthma (n = 12)	Patients with SR asthma (n = 23)	*P* value
Age (y)	49.0 (40.7-57.3)	51.8 (45.9-57.8)	
Ethnic origin			
White	9 (75.0)	17 (73.9)	
African	3 (25.0)	5 (21.7)	
Asian	0	1 (4.4)	
Sex			
Male/female	4 (33.3)/8 (66.6)	14 (60.8)/9 (39.2)	
Atopic∗	10 (83.3)	19 (82.6)	
BMI (kg/m^2^)	31.8 (28.0-35.6)	28.2 (26.2-30.2)	.06
Inhaled corticosteroid dose (BDP)	1113 (798-1469)	1225 (985-1520)	.60
FEV_1_ (L)			
Before steroids	1.7 (1.4-1.9)	1.9 (1.7-2.1)	.13
After steroids	2.1 (1.8-2.4)	1.8 (1.6-2.1)	
FEV_1_ (%)			
Before steroids	56.0 (47.4-64.6)	61.3 (55.3-67.3)	.97
After steroids	70.8 (62.6-79.0)	59.7 (52.8-66.5)	
Serum 25(OH)D (nmol/L)	38.8 (27.6-50.1)	36.9 (27.6-46.2)	.51

Where applicable, data are means with 95% CIs or frequencies (percentages). *P* values are shown for noncategorical clinical parameters assessed by using an unpaired *t* test.

*BDP*, Daily beclomethasone dipropionate equivalent dosage according to the British Thoracic Society/SIGN guidelines on the management of asthma^31^; *BMI*, body mass index.

∗ Atopy was defined based on skin prick test responses.

**Table II:** Cytokine production in cultures from both patients with SS and those with SR asthma at baseline

Cytokine	CD3/IL-2		*P* value	CD3/IL-2 + IL-4		*P* value
Patients with SS asthma	Patients with SR asthma	Patients with SS asthma	Patients with SR asthma
IFN-γ	11.0 (8.3-13.7)	19.7 (14.2-25.2)	.02	8.5 (4.9-12.1)	11.6 (9.0-14.2)	.15
IL-10	1.3 (0.2-2.4)	0.8 (0.5-1.2)	.51	1.2 (0.5-2.0)	1.6 (0.6-2.6)	.61
IL-13	3.6 (0.7-6.6)	2.6 (0.8-4.4)	.89	5.1 (1.7-8.6)	4.3 (2.7-5.8)	.59
IL-17A	11.3 (1.5-21.0)	47.0 (27.8-66.2)	.007	ND	ND	

Cytokine production (in nanograms per milliliter) by cells either stimulated with CD3 plus IL-2 or additionally in the presence of IL-4 in culture is shown. Data are means and 95% CIs.

*ND*, Not detected/measured.

## References

[1] Martinez F.D., Vercelli D. (2013). Asthma. Lancet.

[2] Wenzel S.E. (2012). Asthma phenotypes: the evolution from clinical to molecular approaches. Nat Med.

[3] Chung K.F., Wenzel S.E., Brozek J.L., Bush A., Castro M., Sterk P.J. (2014). International ERS/ATS guidelines on definition, evaluation and treatment of severe asthma. Eur Respir J.

[4] Wenzel S.E., Balzar S., Ampleford E., Hawkins G.A., Busse W.W., Calhoun W.J. (2007). IL4R alpha mutations are associated with asthma exacerbations and mast cell/IgE expression. Am J Respir Crit Care Med.

[5] Wenzel S., Wilbraham D., Fuller R., Getz E.B., Longphre M. (2007). Effect of an interleukin-4 variant on late phase asthmatic response to allergen challenge in asthmatic patients: results of two phase 2a studies. Lancet.

[6] Laviolette M., Gossage D.L., Gauvreau G., Leigh R., Olivenstein R., Katial R. (2013). Effects of benralizumab on airway eosinophils in asthmatic patients with sputum eosinophilia. J Allergy Clin Immunol.

[7] Wenzel S., Ford L., Pearlman D., Spector S., Sher L., Skobieranda F. (2013). Dupilumab in persistent asthma with elevated eosinophil levels. N Engl J Med.

[8] Pavord I.D., Korn S., Howarth P., Bleecker E.R., Buhl R., Keene O.N. (2012). Mepolizumab for severe eosinophilic asthma (DREAM): a multicentre, double-blind, placebo-controlled trial. Lancet.

[9] Corren J., Lemanske R.F., Hanania N.A., Korenblat P.E., Parsey M.V., Arron J.R. (2011). Lebrikizumab treatment in adults with asthma. N Engl J Med.

[10] Bullens D.M., Truyen E., Coteur L., Dilissen E., Hellings P.W., Dupont L.J. (2006). IL-17 mRNA in sputum of asthmatic patients: linking T cell driven inflammation and granulocytic influx?. Respir Res.

[11] Al-Ramli W., Prefontaine D., Chouiali F., Martin J.G., Olivenstein R., Lemiere C. (2009). T(H)17-associated cytokines (IL-17A and IL-17F) in severe asthma. J Allergy Clin Immunol.

[12] Agache I., Ciobanu C., Agache C., Anghel M. (2010). Increased serum IL-17 is an independent risk factor for severe asthma. Respir Med.

[13] Molet S., Hamid Q., Davoine F., Nutku E., Taha R., Page N. (2001). IL-17 is increased in asthmatic airways and induces human bronchial fibroblasts to produce cytokines. J Allergy Clin Immunol.

[14] Barczyk A., Pierzchala W., Sozanska E. (2003). Interleukin-17 in sputum correlates with airway hyperresponsiveness to methacholine. Respir Med.

[15] McKinley L., Alcorn J.F., Peterson A., Dupont R.B., Kapadia S., Logar A. (2008). TH17 cells mediate steroid-resistant airway inflammation and airway hyperresponsiveness in mice. J Immunol.

[16] Nanzer A.M., Chambers E.S., Ryanna K., Richards D.F., Black C., Timms P.M. (2013). Enhanced production of IL-17A in patients with severe asthma is inhibited by 1alpha,25-dihydroxyvitamin D3 in a glucocorticoid-independent fashion. J Allergy Clin Immunol.

[17] Li L.B., Leung D.Y., Martin R.J., Goleva E. (2010). Inhibition of histone deacetylase 2 expression by elevated glucocorticoid receptor beta in steroid-resistant asthma. Am J Respir Crit Care Med.

[18] Vazquez-Tello A., Semlali A., Chakir J., Martin J.G., Leung D.Y., Eidelman D.H. (2010). Induction of glucocorticoid receptor-beta expression in epithelial cells of asthmatic airways by T-helper type 17 cytokines. Clin Exp Allergy.

[19] Vazquez-Tello A., Halwani R., Hamid Q., Al-Muhsen S. (2013). Glucocorticoid receptor-beta up-regulation and steroid resistance induction by IL-17 and IL-23 cytokine stimulation in peripheral mononuclear cells. J Clin Immunol.

[20] Black P.N., Scragg R. (2005). Relationship between serum 25-hydroxyvitamin d and pulmonary function in the third national health and nutrition examination survey. Chest.

[21] Camargo C.A., Rifas-Shiman S.L., Litonjua A.A., Rich-Edwards J.W., Weiss S.T., Gold D.R. (2007). Maternal intake of vitamin D during pregnancy and risk of recurrent wheeze in children at 3 y of age. Am J Clin Nutr.

[22] Devereux G., Litonjua A.A., Turner S.W., Craig L.C., McNeill G., Martindale S. (2007). Maternal vitamin D intake during pregnancy and early childhood wheezing. Am J Clin Nutr.

[23] Brehm J.M., Schuemann B., Fuhlbrigge A.L., Hollis B.W., Strunk R.C., Zeiger R.S. (2010). Serum vitamin D levels and severe asthma exacerbations in the Childhood Asthma Management Program study. J Allergy Clin Immunol.

[24] Gupta A., Sjoukes A., Richards D., Banya W., Hawrylowicz C., Bush A. (2011). Relationship between serum vitamin D, disease severity, and airway remodeling in children with asthma. Am J Respir Crit Care Med.

[25] Sutherland E.R., Goleva E., Jackson L.P., Stevens A.D., Leung D.Y. (2010). Vitamin D levels, lung function, and steroid response in adult asthma. Am J Respir Crit Care Med.

[26] Wu A.C., Tantisira K., Li L., Fuhlbrigge A.L., Weiss S.T., Litonjua A. (2012). Effect of vitamin D and inhaled corticosteroid treatment on lung function in children. Am J Respir Crit Care Med.

[27] Mann E.H., Chambers E.S., Pfeffer P.E., Hawrylowicz C.M. (2014). Immunoregulatory mechanisms of vitamin D relevant to respiratory health and asthma. Ann N Y Acad Sci.

[28] Hawrylowicz C., Richards D., Loke T.K., Corrigan C., Lee T. (2002). A defect in corticosteroid-induced IL-10 production in T lymphocytes from corticosteroid-resistant asthmatic patients. J Allergy Clin Immunol.

[29] Xystrakis E., Kusumakar S., Boswell S., Peek E., Urry Z., Richards D.F. (2006). Reversing the defective induction of IL-10-secreting regulatory T cells in glucocorticoid-resistant asthma patients. J Clin Invest.

[30] Nanzer A.M., Chambers E.S., Ryanna K., Freeman A.T., Colligan G., Richards D.F. (2014). The effects of calcitriol treatment in glucocorticoid-resistant asthma. J Allergy Clin Immunol.

[31] Levy M.L., Thomas M., Small I., Pearce L., Pinnock H., Stephenson P. (2009). Summary of the 2008 BTS/SIGN British Guideline on the management of asthma. Prim Care Respir J.

[32] Harrington L.E., Hatton R.D., Mangan P.R., Turner H., Murphy T.L., Murphy K.M. (2005). Interleukin 17-producing CD4+ effector T cells develop via a lineage distinct from the T helper type 1 and 2 lineages. Nat Immunol.

[33] Balic A., Harcus Y.M., Taylor M.D., Brombacher F., Maizels R.M. (2006). IL-4R signaling is required to induce IL-10 for the establishment of T(h)2 dominance. Int Immunol.

[34] Little S.A., Chalmers G.W., MacLeod K.J., McSharry C., Thomson N.C. (2000). Non-invasive markers of airway inflammation as predictors of oral steroid responsiveness in asthma. Thorax.

[35] Jeffery L.E., Burke F., Mura M., Zheng Y., Qureshi O.S., Hewison M. (2009). 1,25-Dihydroxyvitamin D3 and IL-2 combine to inhibit T cell production of inflammatory cytokines and promote development of regulatory T cells expressing CTLA-4 and FoxP3. J Immunol.

[36] Barrat F.J., Cua D.J., Boonstra A., Richards D.F., Crain C., Savelkoul H.F. (2002). In vitro generation of interleukin 10-producing regulatory CD4(+) T cells is induced by immunosuppressive drugs and inhibited by T helper type 1 (Th1)- and Th2-inducing cytokines. J Exp Med.

[37] Woodruff P.G., Modrek B., Choy D.F., Jia G., Abbas A.R., Ellwanger A. (2009). T-helper type 2-driven inflammation defines major subphenotypes of asthma. Am J Respir Crit Care Med.

[38] Fletcher J.M., Lonergan R., Costelloe L., Kinsella K., Moran B., O'Farrelly C. (2009). CD39+Foxp3+ regulatory T Cells suppress pathogenic Th17 cells and are impaired in multiple sclerosis. J Immunol.

[39] Chambers E.S., Nanzer A.M., Richards D.F., Ryanna K., Freeman A.T., Timms P.M. (2012). Serum 25-dihydroxyvitamin D levels correlate with CD4(+)Foxp3(+) T-cell numbers in moderate/severe asthma. J Allergy Clin Immunol.

[40] Urry Z., Chambers E.S., Xystrakis E., Dimeloe S., Richards D.F., Gabrysova L. (2012). The role of 1alpha,25-dihydroxyvitamin D3 and cytokines in the promotion of distinct Foxp3+ and IL-10+ CD4+ T cells. Eur J Immunol.

[41] Adcock I.M., Ford P.A., Bhavsar P., Ahmad T., Chung K.F. (2008). Steroid resistance in asthma: mechanisms and treatment options. Curr Allergy Asthma Rep.

[42] Goleva E., Searing D.A., Jackson L.P., Richers B.N., Leung D.Y. (2012). Steroid requirements and immune associations with vitamin D are stronger in children than adults with asthma. J Allergy Clin Immunol.

[43] Leung D.Y., Martin R.J., Szefler S.J., Sher E.R., Ying S., Kay A.B. (1995). Dysregulation of interleukin 4, interleukin 5, and interferon gamma gene expression in steroid-resistant asthma. J Exp Med.

[44] Sousa A.R., Lane S.J., Cidlowski J.A., Staynov D.Z., Lee T.H. (2000). Glucocorticoid resistance in asthma is associated with elevated in vivo expression of the glucocorticoid receptor beta-isoform. J Allergy Clin Immunol.

[45] Ahern P.P., Schiering C., Buonocore S., McGeachy M.J., Cua D.J., Maloy K.J. (2010). Interleukin-23 drives intestinal inflammation through direct activity on T cells. Immunity.

[46] Bending D., De la Pena H., Veldhoen M., Phillips J.M., Uyttenhove C., Stockinger B. (2009). Highly purified Th17 cells from BDC2.5NOD mice convert into Th1-like cells in NOD/SCID recipient mice. J Clin Invest.

[47] Kebir H., Ifergan I., Alvarez J.I., Bernard M., Poirier J., Arbour N. (2009). Preferential recruitment of interferon-gamma-expressing TH17 cells in multiple sclerosis. Ann Neurol.

[48] Jia G., Erickson R.W., Choy D.F., Mosesova S., Wu L.C., Solberg O.D. (2012). Periostin is a systemic biomarker of eosinophilic airway inflammation in asthmatic patients. J Allergy Clin Immunol.

[49] Yosef N., Shalek A.K., Gaublomme J.T., Jin H., Lee Y., Awasthi A. (2013). Dynamic regulatory network controlling TH17 cell differentiation. Nature.

[50] Lange N.E., Litonjua A., Hawrylowicz C.M., Weiss S. (2009). Vitamin D, the immune system and asthma. Exp Rev Clin Immunol.

[51] Peek E.J., Richards D.F., Faith A., Lavender P., Lee T.H., Corrigan C.J. (2005). Interleukin-10-secreting “regulatory” T cells induced by glucocorticoids and beta2-agonists. Am J Respir Cell Mol Biol.

[52] Peters M.C., Mekonnen Z.K., Yuan S., Bhakta N.R., Woodruff P.G., Fahy J.V. (2014). Measures of gene expression in sputum cells can identify TH2-high and TH2-low subtypes of asthma. J Allergy Clin Immunol.

[53] Urry Z., Xystrakis E., Richards D.F., McDonald J., Sattar Z., Cousins D.J. (2009). Ligation of TLR9 induced on human IL-10-secreting Tregs by 1alpha,25-dihydroxyvitamin D3 abrogates regulatory function. J Clin Invest.

[54] Castro M., King T.S., Kunselman S.J., Cabana M.D., Denlinger L., Holguin F. (2014). Effect of vitamin D3 on asthma treatment failures in adults with symptomatic asthma and lower vitamin D levels: the VIDA randomized clinical trial. JAMA.

[55] Urashima M., Segawa T., Okazaki M., Kurihara M., Wada Y., Ida H. (2010). Randomized trial of vitamin D supplementation to prevent seasonal influenza A in schoolchildren. Am J Clin Nutr.

[56] Arshi S., Fallahpour M., Nabavi M., Bemanian M.H., Javad-Mousavi S.A., Nojomi M. (2014). The effects of vitamin D supplementation on airway functions in mild to moderate persistent asthma. Ann Allergy Asthma Immunol.

[57] Yadav M., Mittal K. (2014). Effect of vitamin D supplementation on moderate to severe bronchial asthma. Indian J Pediatr.

[58] Gyetko M.R., Hsu C.H., Wilkinson C.C., Patel S., Young E. (1993). Monocyte 1 alpha-hydroxylase regulation: induction by inflammatory cytokines and suppression by dexamethasone and uremia toxin. J Leukoc Biol.

